# Supplementary material for: Perivascular cell-specific knockout of the stem cell pluripotency gene Oct4 inhibits angiogenesis
Source: Nat Commun. 2019 Feb 27;10:967. doi: 10.1038/s41467-019-08811-z (PMC6393549; doi:10.1038/s41467-019-08811-z)
Supplement: Supplementary file 1 — Supplementary Information [file 41467_2019_8811_MOESM1_ESM.pdf]

Perivascular cell-specific knockout of the stem cell pluripotency gene Oct4 inhibits angiogenesis

Hess et al.

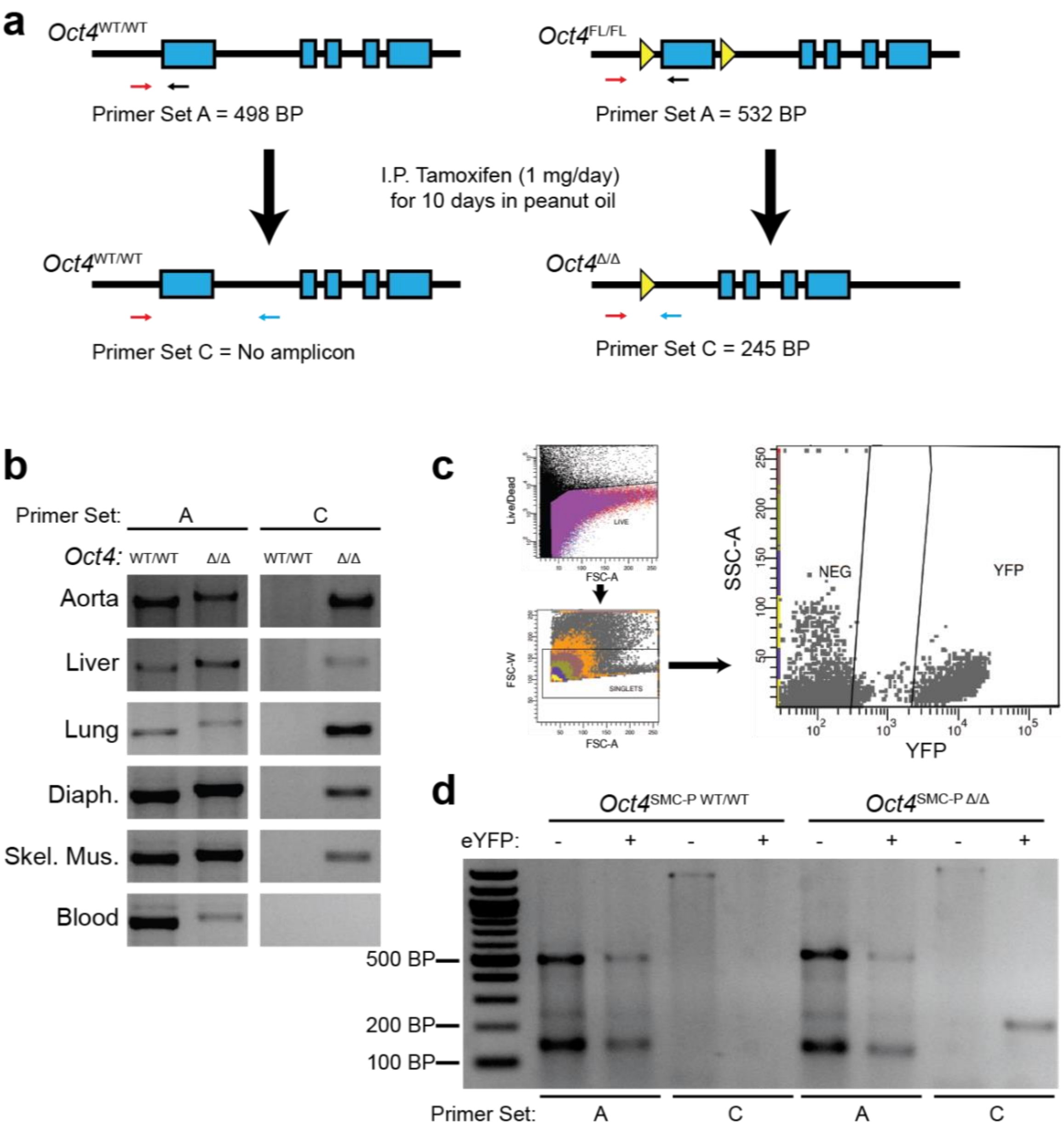

Supplementary Figure 1: Oct4 recombination occurs in multiple tissues of Oct4$^{SMC\text{-}P\ \Delta/\Delta}$ mice. **a**, Primer design for detecting non-recombined (Primer Set A) and recombined (Primer Set C) Oct4 locus. **b**, PCR analysis using primer set A and primer set C on genomic DNA isolated from multiple tissues of Oct4$^{SMC\text{-}P\ WT/WT}$ and Oct4$^{SMC\text{-}P\ \Delta/\Delta}$ mice. **c**, Gating strategy used to sort eYFP- and eYFP+ cells from the calf muscles of Oct4$^{SMC\text{-}P\ WT/WT}$ and Oct4$^{SMC\text{-}P\ \Delta/\Delta}$ mice. d, PCR analysis of sorted cells demonstrates recombination exclusively among eYFP+ cells harvested from Oct4$^{SMC\text{-}P\ \Delta/\Delta}$ mice.

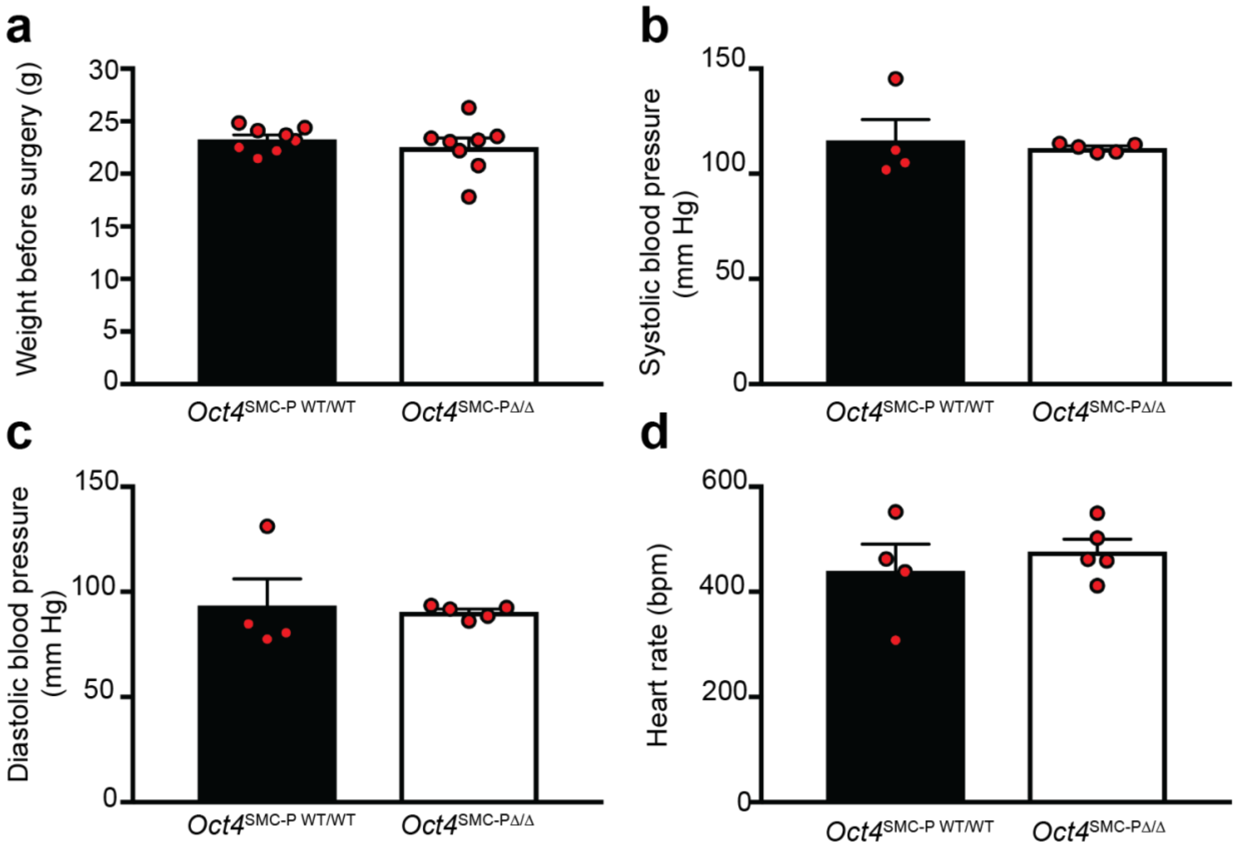

Supplementary Figure 2: **There were no differences in weight, blood pressure, or heart rate following SMC-P Oct4 knockout. a**, Weight of mice immediately prior to surgery (n=8 WT, 8 KO). **b-d**, A catheter-based radiotelemetry system was used to monitor systolic blood pressure (b), diastolic blood pressure (c), and heart rate (d) (n=4 WT, 5 KO). Values = mean ± s.e.m. Statistics were performed using unpaired two-tailed *t*-test (a) or Mann-Whitney *U* test (b-d).

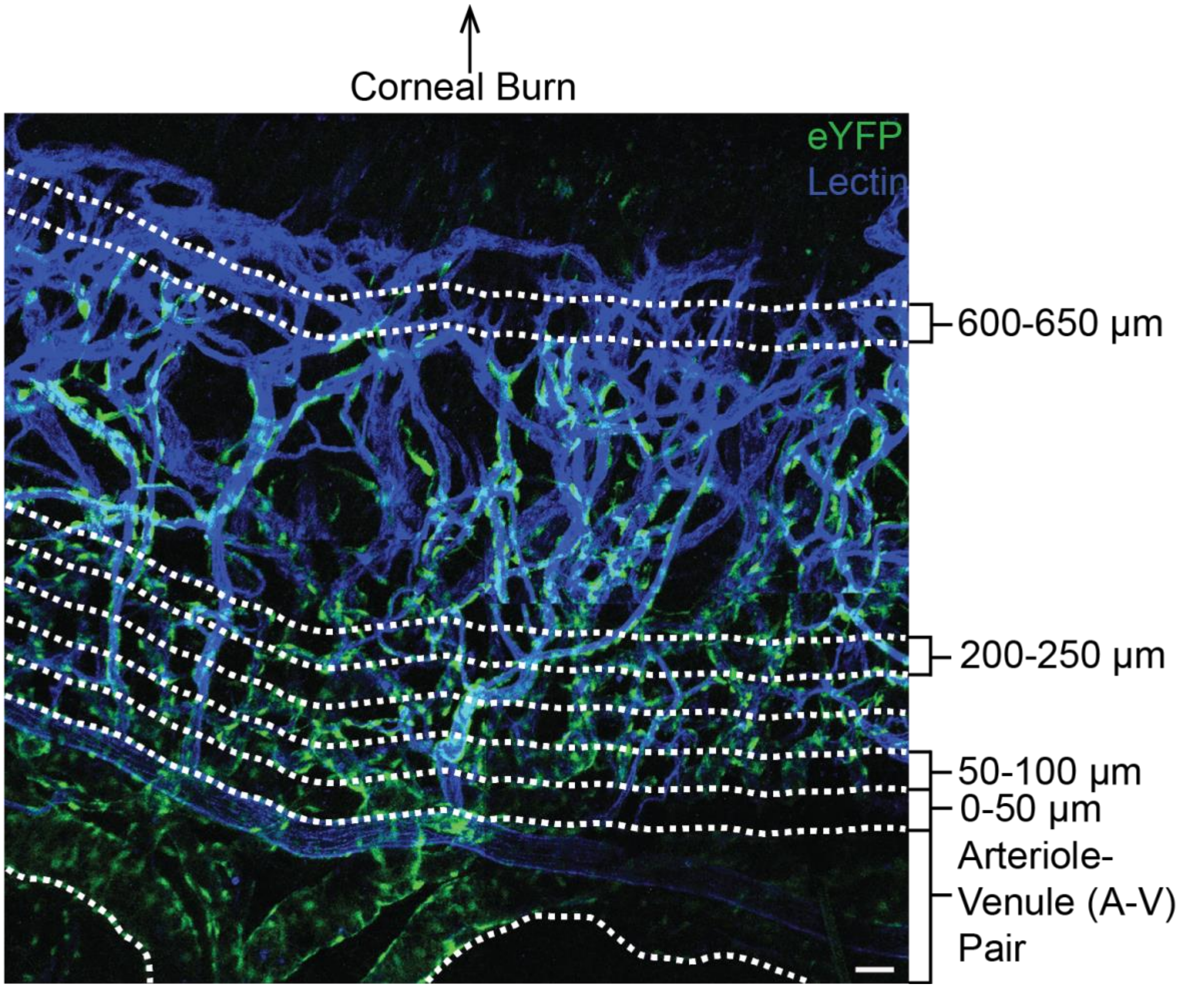

Supplementary Figure 3: Intravital microscopy following corneal burn captures a robust angiogenic response. The area of new growth was divided into 50 μm regions for rigorous quantification of eYFP+ cell density. Shown is a representative montage (several fields of view stitched together) of an Oct4$^{SMC\text{-}P\ WT/WT}$ cornea at day 7 post-corneal burn. eYFP is in green and perfused lectin is in blue. Scale bar = 50 μm.

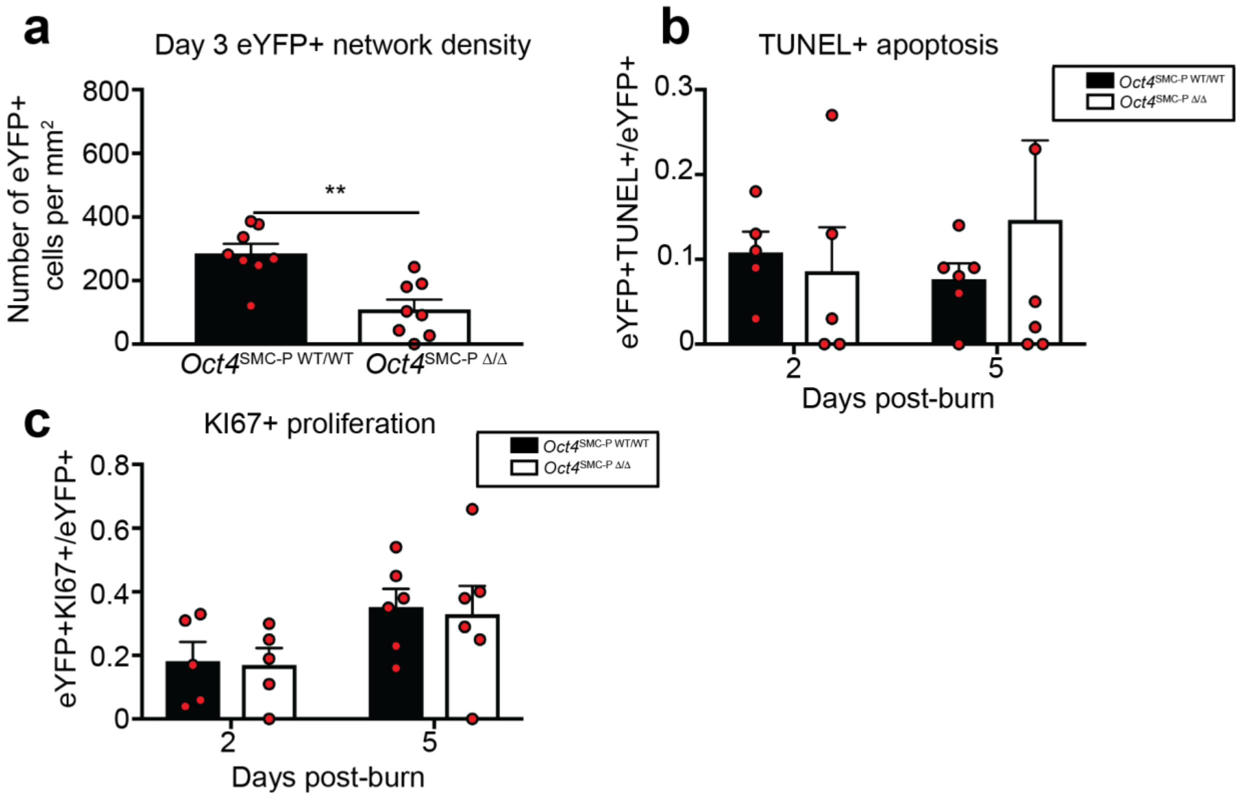

Supplementary Figure 4: SMC-P Oct4 knockout resulted in decreased eYFP+ cell density. **a**, Quantification of eYFP+ cell density of the entire eYFP+ vascular network at day 3 post-burn (n=8 WT, 8 KO). **b**, Quantification of the ratio of eYFP+KI67+ cells to total eYFP+ cells at day 2 (n=5 WT, 5 KO) and day 5 (n=6 WT, 6 KO) post-burn. **c**, Quantification of the ratio of eYFP+TUNEL+ cells to total eYFP+ cells at day 2 (n=5 WT, 5 KO) and day 5 (n=6 WT, 6 KO) post-burn. Values = mean ± s.e.m. Statistics were performed using unpaired two-tailed *t*-test (a, b, c day 2) or Mann-Whitney *U* test (c day 5). $^{**}P < 0.01$.

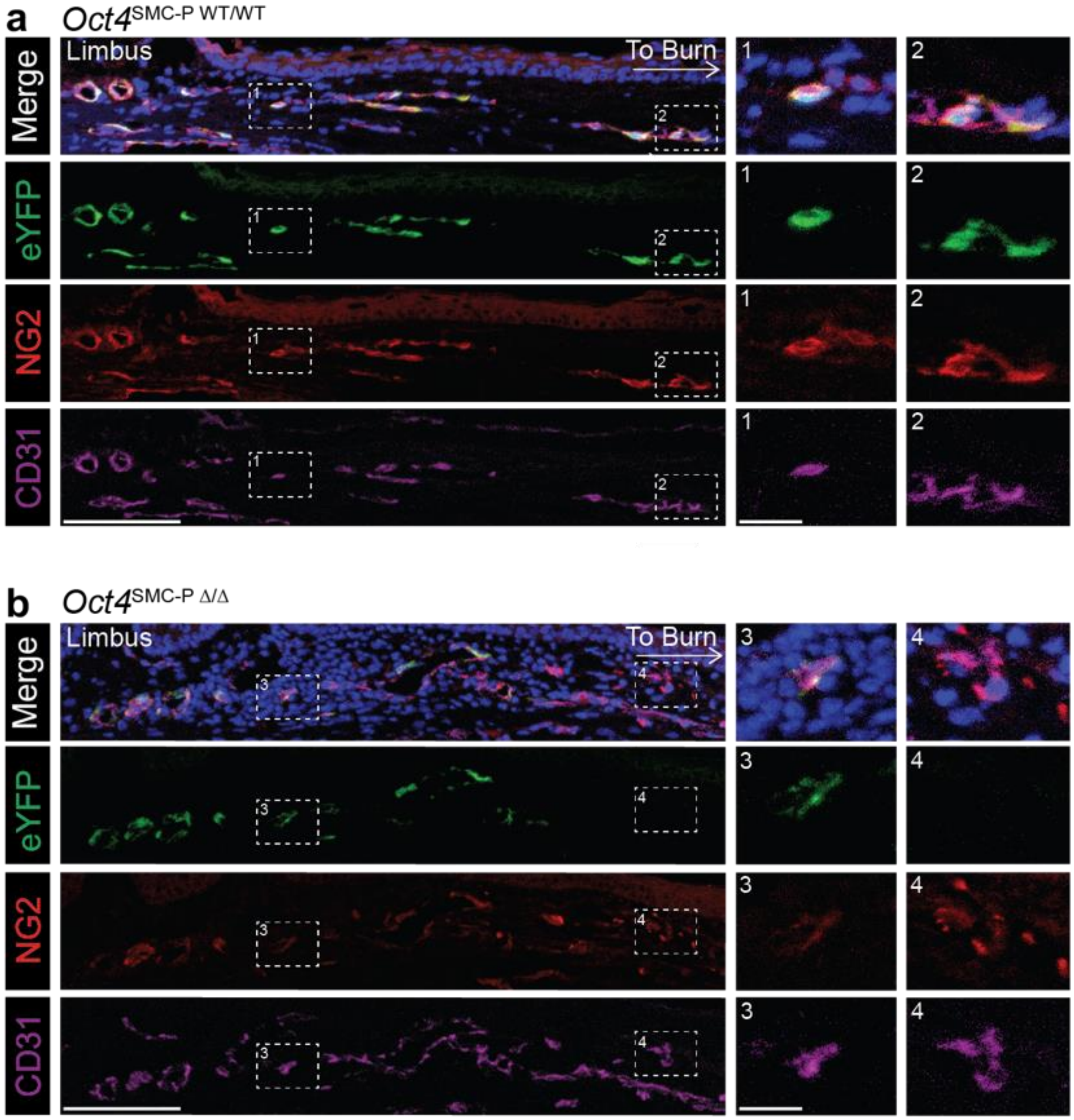

Supplementary Figure 5: Distal CD31+ EC tubes from SMC-P Oct4 knockout corneas were at least partially invested by eYFP-/NG2+ cells. a, Remodeling corneal vasculature from $Oct4^{SMC\text{-}P\ WT/WT}$ mice at day 5 post-corneal burn stained for DAPI, eYFP, NG2, and CD31. Proximal vasculature (box 1) and distal vasculature (box 2) both contain CD31+ cells invested with eYFP+NG2+ cells. **b**, Remodeling corneal vasculature from $Oct4^{SMC\text{-}P\ \Delta/\Delta}$ mice at day 5 post-burn stained for DAPI, eYFP, NG2, and CD31. Proximal vasculature (box 3) contains CD31+ cells invested with eYFP+NG2+ cells. Distal vasculature (box 4) contains CD31+ cells invested with eYFP-/NG2+ cells. n= 3 WT, 3 KO. Representative images are shown. Scale bars = 100 µm, 20 µm (zoom-in regions).

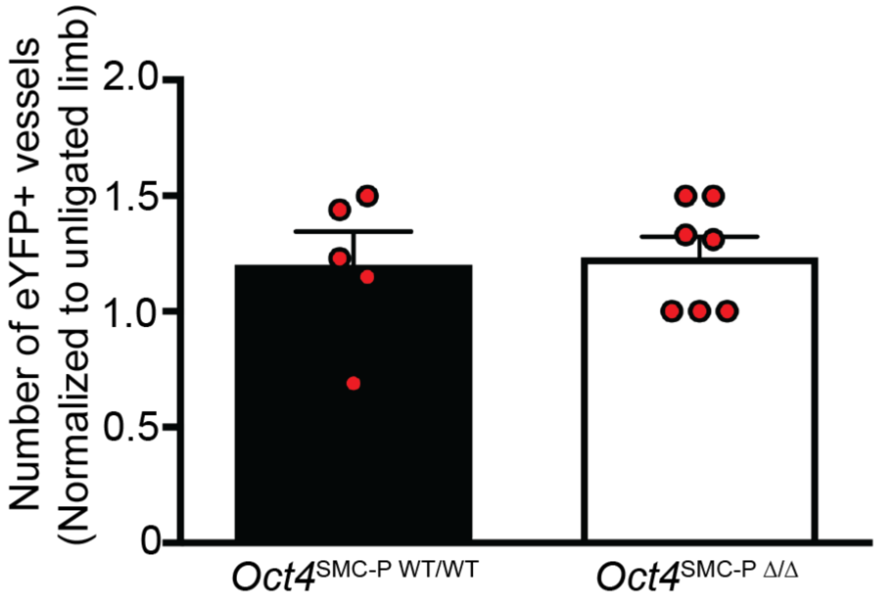

Supplementary Figure 6: SMC-P Oct4 knockout did not affect arteriogenesis following HLI. Quantification of the number of eYFP+ vessels > 10μm in diameter in ligated (left) thigh muscle at day 21 post-HLI normalized to the same parameter in the corresponding unligated (right) thigh muscle (n=5 WT, 7 KO). Values = mean ± s.e.m. Statistics were performed using unpaired two-tailed *t*-test.

270069I18Rik	Hoxc6
Acsbg2	Hoxc9
Adcy8	Il1rapl1
Ano4	Kcnt2
Ano5	Klhl6
Asxl3	Lphn3
Cdh8	Mef2c
Ctsk	Nrap
Dio2	Olfr1317
Dlg2	Olfr1318
Ebf1	Ppp1r9a
Elavl2	Prrt1
Etv1	Robo2
Gm10664	Slit3
Grm7	Sp8
Hoxa11	Sv2c
Hoxa11as	Tox
Hoxa7	Vwc2l
Hoxc4	Zfp385b

Supplementary Table 1: Differentially expressed genes and putative Oct4 targets. A list of the 38 genes that were both differentially regulated in cultured Oct4$^{WT/WT}$ and Oct4$^{\Delta/\Delta}$ SMC treated with hypoxia (1% O_2) as well as putative Oct4 target genes using the oPOSSUM database.
