# Supplementary material for: Perivascular cell-specific knockout of the stem cell pluripotency gene Oct4 inhibits angiogenesis
Source: Nat Commun. 2019 Feb 27;10:967. doi: 10.1038/s41467-019-08811-z (PMC6393549; doi:10.1038/s41467-019-08811-z)
Supplement: Supplementary file 2 — Description of Additional Supplementary Files [file 41467_2019_8811_MOESM2_ESM.docx]

**Description of Supplementary Files**

**File Name:** Supplementary Movie 1

**Description:** 70kDa rhodamine dextran remained mostly confined to the vasculature at day 3 post-burn in Oct4SMC-P WT/WT corneas. 70 kDa rhodamine dextran was injected retro-orbitally immediately (within five minutes) prior to live confocal imaging of a single field of view of an Oct4SMC-PWT/WT cornea for 90 minutes, followed by volume rendering and conversion to movie files.

**File Name:** Supplementary Movie 2

**Description:** 70 kDa rhodamine dextran leaked extensively into tissue parenchyma at day 3 post-burn in Oct4SMC-P ∆/∆ corneas. 70 kDa rhodamine dextran was injected retro-orbitally immediately prior to live confocal imaging of a single field of view of an Oct4SMC-P ∆/∆ cornea for 90 minutes, followed by volume rendering and conversion to movie files.
