# Supplementary material for: Perivascular cell-specific knockout of the stem cell pluripotency gene Oct4 inhibits angiogenesis
Source: Nat Commun. 2019 Feb 27;10:967. doi: 10.1038/s41467-019-08811-z (PMC6393549; doi:10.1038/s41467-019-08811-z)
Supplement: Supplementary file 5 — Reporting Summary [file 41467_2019_8811_MOESM5_ESM.pdf]

nature research

Corresponding author(s): Gary K. Owens

Last updated by author(s): Dec 20, 2018

Reporting Summary

Nature Research wishes to improve the reproducibility of the work that we publish. This form provides structure for consistency and transparency in reporting. For further information on Nature Research policies, see Authors & Referees and the Editorial Policy Checklist.

Statistics

For all statistical analyses, confirm that the following items are present in the figure legend, table legend, main text, or Methods section.

n/a	Confirmed	
☐	☒	The exact sample size (*n*) for each experimental group/condition, given as a discrete number and unit of measurement
☐	☒	A statement on whether measurements were taken from distinct samples or whether the same sample was measured repeatedly
☐	☒	The statistical test(s) used AND whether they are one- or two-sided<br>*Only common tests should be described solely by name; describe more complex techniques in the Methods section.*
☒	☐	A description of all covariates tested
☐	☒	A description of any assumptions or corrections, such as tests of normality and adjustment for multiple comparisons
☐	☒	A full description of the statistical parameters including central tendency (e.g. means) or other basic estimates (e.g. regression coefficient) AND variation (e.g. standard deviation) or associated estimates of uncertainty (e.g. confidence intervals)
☐	☒	For null hypothesis testing, the test statistic (e.g. *F*, *t*, *r*) with confidence intervals, effect sizes, degrees of freedom and *P* value noted<br>*Give P values as exact values whenever suitable.*
☒	☐	For Bayesian analysis, information on the choice of priors and Markov chain Monte Carlo settings
☒	☐	For hierarchical and complex designs, identification of the appropriate level for tests and full reporting of outcomes
☒	☐	Estimates of effect sizes (e.g. Cohen's *d*, Pearson's *r*), indicating how they were calculated

Our web collection on statistics for biologists contains articles on many of the points above.

Software and code

Policy information about availability of computer code

Data collection	Immunofluorescent staining was imaged using a Zeiss LSM700 confocal microscope. Intravital microscopy was performed on a Nikon TE200-E2 confocal micoscope.
Data analysis	Zen 2009 Light Edition Software (Zeiss) was used for analysis of immunofluorescent z-stack images and single cell counting. Pixel density of immunofluorescent images was analyzed with Image Pro Plus Software 7.0 (Media Cybernetics). Analysis of intravital microscopy images was performed using Fiji (ImageJ). MATLAB was used to generate movie files. Analysis of scratch wound assays was performed using the cell counter plugin on ImageJ.

For manuscripts utilizing custom algorithms or software that are central to the research but not yet described in published literature, software must be made available to editors/reviewers. We strongly encourage code deposition in a community repository (e.g. GitHub). See the Nature Research guidelines for submitting code & software for further information.

Data

Policy information about availability of data

All manuscripts must include a data availability statement. This statement should provide the following information, where applicable:
- Accession codes, unique identifiers, or web links for publicly available datasets
- A list of figures that have associated raw data
- A description of any restrictions on data availability

The data that supports this study is available from the corresponding author upon request.

Field-specific reporting

Please select the one below that is the best fit for your research. If you are not sure, read the appropriate sections before making your selection.

☒ Life sciences ☐ Behavioural & social sciences ☐ Ecological, evolutionary & environmental sciences

For a reference copy of the document with all sections, see nature.com/documents/nr-reporting-summary-flat.pdf

Life sciences study design

All studies must disclose on these points even when the disclosure is negative.

Sample size	Sample size was chosen based on previous studies (Cherepanova et al., Nat Med, 2016).
Data exclusions	Data was first tested for normality. If normally distributed, Grubb's outlier test was used to test for outliers. Any outlier was excluded from analysis.
Replication	Attempts at replication were successful.
Randomization	For all Oct4 KO studies, we utilized Oct4wt/wt and Oct4fl/fl littermates to ensure that the Oct4 WT and KO animals were genetically identical with exception of exon 1 of the Oct4 locus. All mice were on a C57Bl/6 background and had been backcrossed for at least 9 generations.<br><br>The location of the Myh11 Cre ERT2 transgene on the Y chromosome precludes the use of females in this study.
Blinding	Researchers were blinded to the genotype of the animals during data acquisition and analysis.

Reporting for specific materials, systems and methods

We require information from authors about some types of materials, experimental systems and methods used in many studies. Here, indicate whether each material, system or method listed is relevant to your study. If you are not sure if a list item applies to your research, read the appropriate section before selecting a response.

Materials & experimental systems

n/a	Involved in the study
☐	☒ Antibodies
☐	☒ Eukaryotic cell lines
☒	☐ Palaeontology
☐	☒ Animals and other organisms
☒	☐ Human research participants
☒	☐ Clinical data

Methods

n/a	Involved in the study
☒	☐ ChIP-seq
☒	☐ Flow cytometry
☒	☐ MRI-based neuroimaging

Antibodies

Antibodies used

For immunofluorescent staining, the following antibodies were used:

Goat polyclonal GFP (Abcam, ab6673, 1:100)
Rat CD31 (Dianova, DIA-310 (Clone SZ31, 1:250)
Rabbit Slit3 (Sigma, SAB2104337, 1:50).
Rabbit KI67 (Abcam, ab15583, 1:500)
Rabbit NG2 Chondroitin Sulfate Proteoglycan (Millipore, AB5320, 1:500)
Rabbit PDGFRB (Abcam, ab32570, 1:500)

The following secondary antibodies were used:

Donkey anti-goat alexa 488 (Invitrogen, A11055, 1:250)
Donkey anti-goat alexa 555 (Invitrogen, A21432, 1:250)
Donkey anti-goat alexa 647 (Invitrogen, A21447, 1:250)
Donkey anti-rabbit alexa 488 (Invitrogen, A21206, 1:250)
Donkey anti-rabbit alexa 555 (Invitrogen, A31572, 1:250)
Donkey anti-rat conjugated to Dylight 550 (Abcam, ab102261, 1:250)
Donkey anti-rat conjugated to Dylight 650 (Abcam, ab102263, 1:250)

Validation

Antibody specificity was determined by using an IgG isotype control raised in the same species as the primary antibody.

Optimization was performed by testing a range of dilutions of each antibody and IgG control.

Eukaryotic cell lines

Policy information about cell lines

Cell line source(s)	Mouse aortic SMC were isolated from thoracic aortas of 6-week-old male Oct4 SMC-P WT/WT and Oct4 SMC-P Δ/Δ mice, after tamoxifen injections. Mouse aortic endothelial cells were purchased from Cell Biologics (Catalog No. C57-6052).
Authentication	Mouse aortic SMC were authenticated as previously described (Cherepanova, Nat Med, 2016).
Mycoplasma contamination	Cell lines were not tested for mycoplasma contamination.
Commonly misidentified lines (See ICLAC register)	N/A

Animals and other organisms

Policy information about studies involving animals; ARRIVE guidelines recommended for reporting animal research

Laboratory animals	Myh11-CreERT2 ROSA floxed STOP eYFP Oct4WT/WT and Myh11-CreERT2 ROSA floxed STOP eYFP Oct4FL/FL male littermate mice were used for experiments, which were genetically identical other than containing WT versus floxed Oct4 alleles. We only used male progeny for experiments, since the Myh11-CreERT2 transgene is located on the Y chromosome. Upon tamoxifen treatment, there is simultaneous activation of eYFP and excision of the floxed Oct4 alleles, generating Myh11-CreERT2 ROSA eYFP Oct4WT/WT and Myh11-CreERT2 ROSA eYFP Oct4Δ/Δ mice. At 12-18 weeks of age, mice were used for experiments.
Wild animals	The study did not involve wild animals.
Field-collected samples	The study did not involve samples collected from the field.
Ethics oversight	All animal protocols were approved by and overseen by the University of Virginia Animal Care and Use Committee.

Note that full information on the approval of the study protocol must also be provided in the manuscript.
